# P063. Colloid cysts of the third ventricle: a case report

**DOI:** 10.1186/1129-2377-16-S1-A131

**Published:** 2015-09-28

**Authors:** Silvia de Pasqua, Valentina Favoni, Giulia Giannini, Rossana Terlizzi, Giulia Pierangeli, Pietro Cortelli, Sabina Cevoli

**Affiliations:** Department of Biomedical and NeuroMotor Sciences (DIBINEM), Alma Mater Studiorum-University of Bologna, IRCCS Institute of Neurological Sciences of Bologna, Bologna, Italy

## Background

Colloid cysts are rare congenital benign tumors accounting for 0.2-2% of all intracranial neoplasms. They usually occur in the front part of the third ventricle. The clinical presentation is related to the increased intracranial pressure and is widely variable; sudden death associated with acute hydrocephalus can occur, therefore recognition of this rare condition is important in order to select an appropriate surgical treatment. We report a case of new onset headache secondary to a colloid cyst of the third ventricle.

## Case report

A 44-year-old woman with unremarkable medical history was admitted to our clinic for recurrent attacks of pressing headache, with abrupt onset and brief duration, accompanied by nausea, transient hearing loss and tinnitus. These symptoms were relieved by supine position. The clinical picture progressively worsened, with episodes of vomiting during headache. Neurological examination was negative. A magnetic resonance imaging (MRI) scan showed a spherical mass lesion with lipid signal at the intraventricular foramina of Monro causing compression of the third ventricle and expansion of the ventricular system, suggestive of hydrocephalus.

After neurosurgical evaluation the patient underwent an endoscopic removal of the lesion. Histological findings were compatible with colloid cyst of the third ventricle. After surgical treatment the patient recovered completely from symptoms and the follow-up MRI demonstrated the complete excision of the lesion.

## Conclusions

Colloid cysts are congenital, slow growing, benign intraventricular lesions usually arising in the third ventricle. They may cause obstruction of the foramen of Monro with blockage of cerebrospinal fluid (CSF) flow, producing progressive or intermittent elevated intracranial pressure with chronic or acute hydrocephalus [1]. The onset of symptoms is usually between 20 to 50 years of age, often with paroxismal attacks of severe headache associated with nausea and vomiting [2]. Presentations with thunderclap headache have also been described [3]. Headache can resolve or reduce in supine position, suggesting that the colloid cyst moves in and out of the foramen of Monro with intermittent obstruction of CSF. Colloid cysts, if left untreated, may lead to serious complications such as visual loss, memory difficulties, acute disturbances of consciousness and even sudden death as a consequence of acute obstructive hydrocephalus with brain herniation or cardiovascular failure due to abrupt disturbance of hypothalamic function. The risk of sudden neurologic deterioration cannot be predicted, therefore surgical treatment is recommended [1, 2].

Our case highlights the importance of early detection and prompt treatment of this potentially life-threatening cause of headache.

Written informed consent to publish was obtained from the patient(s).
